# Clustering analysis of circulating plasma-biomarkers in atopic dermatitis reveals a distinct-inflammatory cardiac risk profile in African American patients

**DOI:** 10.21203/rs.3.rs-7862416/v1

**Published:** 2025-12-01

**Authors:** Davies Gage, Emily Z. Ma, Deena Fayyad, Yagiz M. Akiska, Shahin Shahsavari, Aaron Bao, Magdi Elghannam, Perya Bhagchandani, Shabnam Afzal, Waleed Adawi, Kristin L. Khan, Manu M. Mysore, Shawn G. Kwatra

**Affiliations:** University of Maryland, Baltimore; University of Maryland, Baltimore; University of Maryland, Baltimore; University of Maryland, Baltimore; University of Maryland, Baltimore; University of Maryland, Baltimore; University of Maryland, Baltimore; University of Maryland, Baltimore; University of Maryland, Baltimore; University of Maryland, Baltimore; University of Maryland, Baltimore; University of Maryland, Baltimore; University of Maryland, Baltimore

**Keywords:** Atopic dermatitis, African Americans, Cardiovascular diseases, Pruritus, Chronic itch

## Abstract

Atopic dermatitis (AD) is a heterogeneous inflammatory skin disease associated with increased cardiovascular risk, disproportionately affecting African Americans (AA). However, AA patients’ cardiac risk profile in AD remains unclear. Plasma from 42 moderate-to-severe AD patients (worst itch numeric rating scale [WI-NRS] ≥ 7, investigator global assessment scale [IGA] ≥ 3) and 37 matched healthy controls (HC) were assayed for 9 cardiovascular disease proteins. A multi-center analysis was performed to compare cardiovascular comorbidities and inflammatory biomarkers racially. African American AD patients had higher C-reactive protein (CRP) (30.9 mg/L AA vs. 23.8 HC), positively correlating with WI-NRS (r = 0.34). Hierarchical clustering identified two clusters: Cluster 2 (80% AA, n = 4) showing elevated CRP (160 mg/mL vs. 10), haptoglobin (532.2 mg/dL vs. 55.8), α1-acid glycoprotein (0.11 g/dL vs. 0.08), and serum amyloid P (0.48 mg/dL vs. 0.32) compared to Cluster 1 (59% AA, n = 22). Multi-center data revealed AA AD patients had increased risk of ischemic heart disease, hypertension, and atherosclerosis than Caucasian Americans (CA) AD patients, along with elevated pro-inflammatory markers (CRP, ferritin, ESR, and eosinophils). The study’s cross-sectional retrospective design prevents establishing causality. Our results highlight an increased cardiac risk and systemic inflammatory profile in AA patients with AD.

## Introduction

Atopic dermatitis (AD) is a heterogenous inflammatory skin disease that often begins in infancy though it may present later in life [1].The hallmark symptoms of this disease, intense itch and pain, severely impacts quality of life and disrupts sleep leading to psychological distress [2]. AD has been associated with increased risk of numerous systemic conditions including hematopoietic malignancies, metabolic syndrome, autoimmune diseases and cardiovascular diseases [3–5].

The literature suggests that the immunologic profiles are not uniform but vary across racial and ethnic groups highlighting the diseases heterogeneity [6]. African American (AA) patients are disproportionally affected by AD when compared to Caucasian (CA) patients [7 = 8]. AA patients with AD have been shown to have different immune profiles, genetic risk profiles, greater disease burden, often experience delayed diagnosis, and increased risk for developing more severe forms of AD [9–13].

Higher disease burden and increased AD severity had been associated with increased cardiovascular outcomes in patients [4]. AA AD patients are already known to vary when compared to CA patients in regard to AD severity and cardiovascular risk, however the cardiac risk profile in AA with AD remains understudied [14]. The association of AD with systemic comorbidities and racial differences suggests the possibility for disease endotypes that can be targeted.

In our study we aim to characterize cardiac biomarkers in AA patients with AD by comparing inflammatory cytokine levels and risk for cardiovascular comorbidities in patients with AD in a multicenter cohort study. We hypothesize that unique cytokine and cardiac risk profiles exist in subpopulations of patients with AD.

## Methods

### Recruited patients

This study was approved by the Johns Hopkins Institutional Review Board (IRB00119007). A prospective study was performed comparing circulating plasma biomarkers from 42 adult patients with moderate-to-severe AD and 37 healthy controls (HCs) matched for age, race, and sex.

Inclusion criteria for AD patients were defined as a diagnosis by a board-certified dermatologist, a worst itch numeric rating scale (WI-NRS) score of ≥ 7, and an Investigator’s Global Assessment (IGA) score of ≥ 3. Self-reported demographic and measured clinical characteristics of patients were collected at the time of blood draw.

### Blood processing and cytokine quantification

Plasma samples were analyzed for the presence of human cardiovascular markers. Multiplexed quantification was conducted using the Luminex^™^ 200 system (Luminex, Austin, TX, USA) by Eve Technologies Corp (Calgary, Alberta) utilizing the Human Cardiovascular Disease Panel 3 9-Plex Discovery Assay^®^ (MilliporeSigma, Burlington, MA, USA), according to the manufacturer’s protocol. The 9-plex included α-2 Macroglobulin, AGP, CRP, Fetuin A, Fibrinogen, Haptoglobin, L-Selectin, PF4, and SAP, with assay sensitivities ranging from 1 to 89 pg/mL. as detailed in the MilliporeSigma MILLIPLEX^®^ MAP protocol. All samples were measured in 96-well plates, and data were analyzed following the standard curves with a four-parameter logistic fit using the manufacturer’s software.

### Population-level analysis

Population-level data was queried from TriNetX, a global federated health research network aggregating electronic medical records from 97 healthcare organizations, with data spanning from 2005–2023. The analysis compared two cohorts of adult patients diagnosed with atopic dermatitis (AD): Black or African American (Cohort 1, AA AD) and White (Cohort 2, CA AD). Inclusion criteria were defined as ≥ 2 recorded diagnoses of AD (ICD-10-CM: L20) and age ≥ 18 years at the time of the most recent AD diagnosis. TriNetX race data was queried through electronic medical record data and coded to HL7 administrative standards.

Propensity score matching (PSM) was performed to address confounding and balance baseline demographic and clinical characteristics between groups. A logistic regression model calculated propensity scores based on age, sex, and baseline comorbidities including allergic rhinitis (ICD-10-CM: J30.9) and asthma (ICD-10-CM: J45). Patients were matched 1:1 without replacement using a nearest-neighbor algorithm with a caliper width of 0.2 of the standard deviation of the logit of the propensity score. Standardized mean differences (SMDs) were calculated to ensure covariate balance, with all SMDs reduced to < 0.1 after matching.

The primary outcomes were defined as laboratory markers of systemic inflammation (C-reactive protein [CRP], ferritin, erythrocyte sedimentation rate [ESR], eosinophil counts), as well as cardiovascular comorbidities (hypertension [HTN], ischemic heart disease [IHD], atherosclerosis, stroke, myocardial infarction [MI], peripheral vascular disease [PVD]). Laboratory outcomes were analyzed as continuous variables, while cardiovascular outcomes were assessed as binary variables.

### Statistical Analysis

For the TriNetX cohort, continuous outcomes were analyzed using two-tailed t-tests, with results reported as mean ± standard deviation. Binary outcomes were evaluated by calculating risk differences, risk ratios, and odds ratios. Statistical significance was defined as P < 0.05. All analyses were performed using web-based TriNetX platform. The data were de-identified, and the study complied with ethical guidelines for secondary data use.

For the recruited patient cohort, two tailed t-tests were used to compare plasma inflammatory biomarker levels across groups, while risk ratios with 95% confidence intervals were calculated to assess the risk of developing cardiac comorbidities across groups. We examined the correlation between WI-NRS itch severity scores and plasma cytokine levels using Pearson’s correlation test.

## Results

### TrinetX Cohorts

#### Demographic Characteristics

Cohort 1 included African American patients, while Cohort 2 included Caucasian patients. Initial cohort sizes were 43,695 and 86,263 for Cohorts 1 and 2, respectively. After propensity score matching, both cohorts included 37,494 patients, with balanced demographic and clinical characteristics. The mean age in the AA patient cohort was 28.6 years (SD: 21.2), compared to 28.8 years (SD: 21.1) in the CA patient cohort (Table 1). Female representation was similar across cohorts, with 65.1% in the AA patient cohort and 65.7% in the CA patient cohort (Table 1). The prevalence of allergic rhinitis was slightly lower in the AA cohort (16.6%) than in the CA cohort (17.1%), while asthma prevalence was comparable (23.3% in the AA cohort versus 23.5% in the CA cohort) (Table 1). None of these differences were statistically significant (P > 0.05), indicating effective matching of baseline characteristics (Table 1).

### Laboratory Outcomes

Significant differences in laboratory markers were observed between the matched cohorts. Mean CRP levels were higher in the AA patient cohort (24.50 mg/L; SD: 48.07) compared to the CA cohort (17.95 mg/L; SD: 38.12; P < 0.001). Similarly, ferritin levels were elevated in the matched AA cohort (206.62 ng/mL; SD: 966.35) compared to CA cohort (128.47 ng/mL; SD: 448.49; P < 0.001). ESR was also higher in AA cohort (28.21 mm/hr; SD: 25.41) relative to CA cohort (15.48 mm/hr; SD: 17.74; P < 0.001).

Eosinophil counts were elevated in AA cohort, with a mean of 1.24 10*3/uL (SD: 8.70) compared to 0.83 10*3/uL (SD: 6.95) in CA cohort (P < 0.001).

### Clinical Outcomes

**Figure 1:**
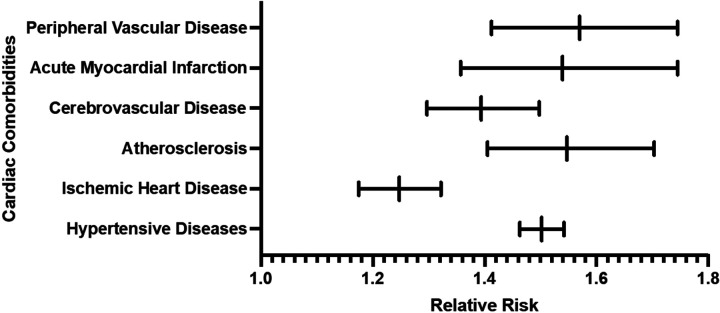
Comparative risk of cardiovascular comorbidities between matched African American and Caucasian patients with atopic dermatitis.

The risk of HTN was significantly higher in the AA patient cohort, with 28.3% of patients affected compared to 18.9% in CA cohort (RR: 1.50, 95% CI: 1.46–1.54, P < 0.001) (Fig. 1). Similarly, IHD was more prevalent in the AA cohort (6.2%) than in CA cohort 2 (5.0%) (RR: 1.25, 95% CI: 1.18–1.32, P < 0.001) (Fig. 1). The AA cohort also exhibited increased risks for atherosclerosis (RR: 1.55, 95% CI: 1.41–1.70), stroke (RR: 1.39, 95% CI: 1.30–1.50), MI (RR: 1.54, 95% CI: 1.36–1.75), and PVD (RR: 1.57, 95% CI: 1.41–1.75), all P < 0.001 (Fig. 1). Kaplan-Meier survival analyses confirmed higher cumulative risks for these outcomes in the AA patient cohort, with statistically significant differences in survival probability across all measured conditions.

### Our cohort

**Figure 2:**
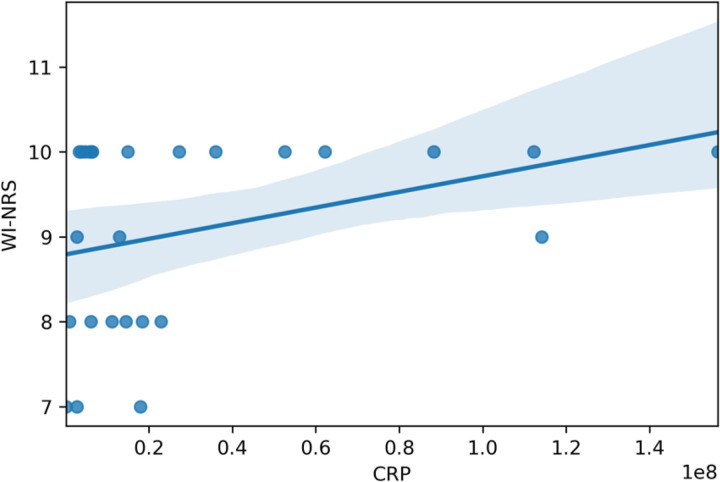
Spearman correlation between CRP levels and worst itch numeric rating scale among African American patients with Atopic Dermatitis (r=0.35, p<0.05).

**Figure 3:**
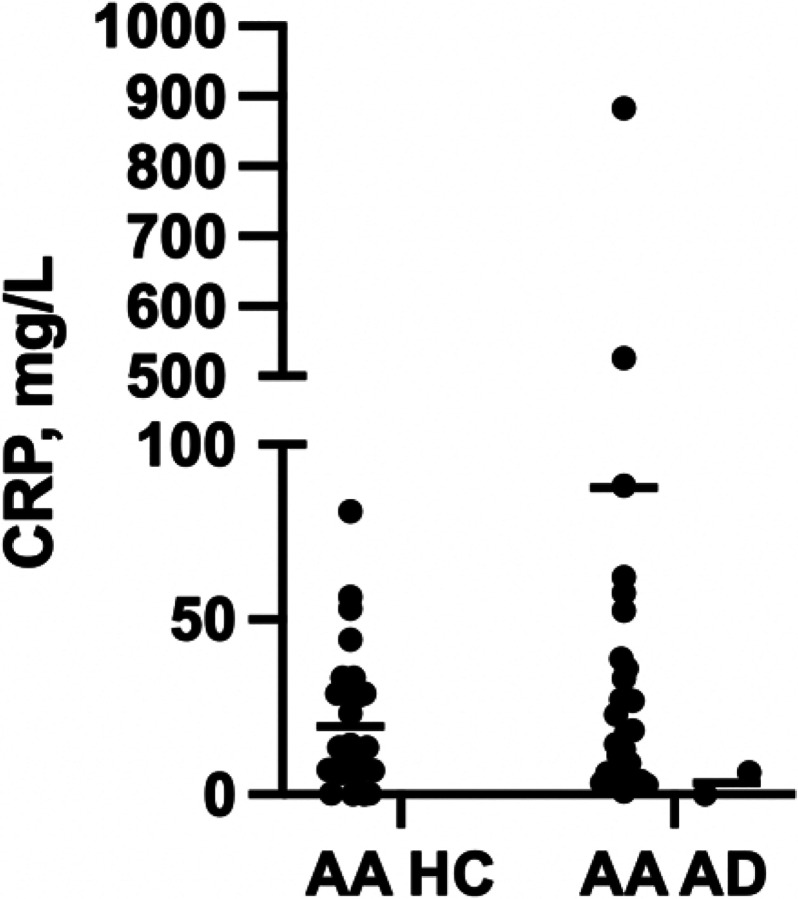
CRP levels in AA HC vs. AA AD patients. AA AD patients exhibited significantly increased levels of CRP in contrast to their controls (p=0.02). AA, African American; HC, healthy controls; AD, atopic dermatitis.

**Figure 4:**
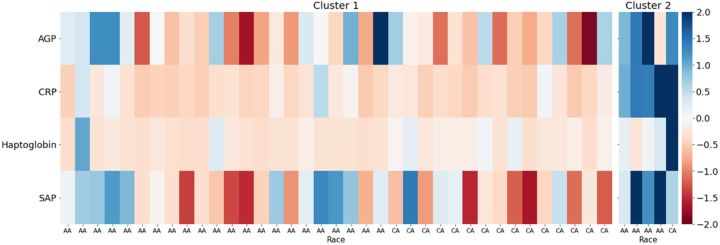
Hierarchical clustering of atopic dermatitis patients: Heatmap of z-score transformed biomarker levels for each patient, delineated by cluster. Biomarkers were grouped with agglomerative hierarchical clustering. AA, African American; CA, Caucasian; AGP, α1-acid glycoprotein; SAP, serum amyloid P component.

Significant differences in CRP levels were observed with AA AD patients having higher C-reactive protein (CRP) (30.9 mg/L (n = 26) vs. 23.8 (HC)(n = 18), p = 0.02) (Fig. 2). CRP was also positively correlated with WI-NRS in AA AD patients (r = 0.34, p < 0.05) (Fig. 3). Using hierarchical clustering, we identified two distinct clusters of AD patients (Cluster 1, n = 37, Cluster 2, n = 5) with Cluster 2 composed of more AA patients compared to Cluster 1 (59%, n = 22 vs. 80%, n = 4). Cluster 2 had higher levels of cardiac proteins CRP (Cluster 1 = 10 mg/mL vs. Cluster 2 = 160), haptoglobin (55.8 mg/dL vs. 532.2), α1-acid glycoprotein (0.08 g/dL vs. 0.11), and serum amyloid P component (0.32 mg/dL vs. 0.48) (all p < 0.05) compared to Cluster 1 (Fig. 4).

## Discussion

AD is a heterogeneous disease characterized by various endotypes and phenotypes [15]. Risk profiles among different patient populations vary and a deeper understanding of associated comorbidities and the molecular profiles driving these characteristics is needed [16–17]. Our study identified several elevated circulating plasma biomarkers in AD that are associated with systemic inflammation. Population-level analysis revealed significantly elevated pro-inflammatory markers in AA AD patients when compared to CA AD patients. Additionally, evaluation of clinical outcomes revealed increased risk of cardiovascular comorbidities among AA AD patients. Hierarchical clustering of recruited patients identified two distinct clusters with different inflammatory profiles. The cluster containing a higher percentage AA patients had a more inflammatory cardiac profile that was additionally associated with itch severity.

It is well-established that AD is associated with increased risk of cardiovascular comorbidities, driven by chronic systemic inflammation [4, 5] .Prior studies have linked AD to increased risk of myocardial infarction, stroke, hypertension, and ischemic heart disease emphasizing the systemic inflammatory burden [4, 5]. Elevated CRP levels have been previously reported in moderate-severe AD, correlating with disease severity, consistent with our findings that CRP levels are associated with Itch severity [3]. Further evidence shows that increased Th2-driven inflammation exacerbates vascular risk and is associated with clinical severity [18].

Our results extend upon this knowledge by highlighting an amplified cardiovascular risk in AA patients with AD, likely driven by differences in inflammatory profiles. Population level analysis and evaluation of our recruited AD cohort revealed elevated pro-inflammatory markers such as CRP, ESR, ferritin, eosinophils and CRP, haptoglobin, α1-acid glycoprotein, and serum amyloid P component respectively in AA AD patients compared to CA patients. Elevated CRP levels have been linked to both increased AD severity and systemic inflammation [19]. CRP, a well-established predictor of cardiovascular events, is particularly relevant among AA populations, who are disproportionately affected by cardiovascular comorbidities and exhibit elevated baseline levels [20]. Our findings also revealed that CRP levels were positively associated with itch severity ratings among AA AD patients corroborating our previous findings that CRP plays an important role in itch, itch severity and associate patient outcomes [21, 22]. Acute phase reactants such as ESR, ferritin, haptoglobin, α1-acid glycoprotein, and serum amyloid P have been associated with various cardiovascular risk factors such as coronary artery disease, coronary plaque volume, the development of heart failure, and increased risk for both MI and stroke, angina and atherosclerosis [23–27]. Eosinophil levels have been shown to have a differential roles in cardiovascular disease (CVD) with prothrombotic and calcific nature chronically but protective in acute contexts [28].

Our clinical findings demonstrate increased risk of cardiovascular conditions among AA AD patients when compared to CA AD patients. The comorbidities with increased risk among AA patients included HTN, IHD, atherosclerosis, stroke, MI, and PVD. Consistent with our findings the risk for CVD among AA populations remain elevated and overall cardiovascular health poor in comparison with CA populations [29–32]. This aligns with broader evidence of poorer cardiovascular health among AA populations and the known association between AD and cardiovascular risk [14]. Our findings suggest a synergistic effect in AA patients, where the inflammatory burden of AD exacerbates pre-existing racial disparities in CVD risk. This is further supported by evidence of elevated Th2 cytokines (e.g., IL-13, IL-4) in AA AD patients demonstrating potential to drive both disease severity and vascular dysfunction [33].

Despite these findings several limitations of our study should be addressed. The cross-sectional and retrospective nature of our study limits the ability to establish causal relationships between our variables thus our results suggest that systemic inflammation related to AD may contribute to the increased cardiovascular risk observed in AA patients. Given their higher prevalence of cardiovascular comorbidities and increased systemic inflammatory markers, our findings emphasize the need for more thorough cardiovascular risk assessments in AA patients with AD. To mitigate long-term risks to cardiovascular health an interdisciplinary approach is ideal between dermatology, primary care, and cardiology. Future research should focus on the impacts of systemic AD treatments on cardiovascular outcomes and known inflammatory markers. Further prospective research with larger populations is needed to explore the underlying mechanisms driving these racial disparities in driving cardiovascular outcomes in AD.

In conclusion, our study builds upon known systemic risk factors within AD by detailing the pro-inflammatory cytokine profiles from a large cohort of AD patients and corroborating our findings with population-level data. Furthermore, this analysis highlights that while AD itself heightens cardiovascular risk, AA patients face an amplified burden, highlighting the importance of race-specific strategies in AD management.

## Figures and Tables

**Table 1:** Baseline demographics of our clinic’s cohort and TriNetX cohorts.

Clinic Cohort				
	AA HC	CA HC	AA AD	CA AD
**Number of Patients**	18	19	26	16
**Age, mean ± SD**	41.9 ± 12.3	48.8 ± 16.7	41.5 ± 16.4	43.6 ± 17.8
**Gender, n (%)**				
**Female**	12 (66.7)	12 (63.2)	16 (61.5)	13 (81.3)
**Male**	6 (33.3)	7 (36.8)	10 (38.5)	3 (18.8)

TrinetX Cohorts						
Characteristic	Before Matching			After Matching		
	Cohort 1 (AA AD)	Cohort 2 (CA AD)	p-value	Cohort 1 (AA AD)	Cohort 2 (CA AD)	p-value
**Number of Patients**	43,695	86,263		37,494	37,494	
**Age, mean ± SD**	28.2 ± 21.1	40.0 ± 23.9	< 0.001	28.6 ± 21.2	28.8 ± 21.1	0.341
**Gender, n (%)**						
Female	25,144 (65.6)	47,003 (59.4)	< 0.001	24,418 (65.1)	24,649 (65.7)	0.076
Male	13,124 (34.3)	32,081 (40.6)	< 0.001	13,044 (34.8)	12,835 (34.2)	0.108
**Co-Diagnoses, n (%)**						
Allergic Rhinitis	6,648 (17.4)	11,128 (14.1)	< 0.001	6,234 (16.6)	6,420 (17.1)	0.07
Asthma	9,499 (24.8)	13,457 (17.0)	< 0.001	8,727 (23.3)	8,811 (23.5)	0.469

## Data Availability

The data generated and analyzed in this study are available from the corresponding author upon reasonable request.

## What is already known about this topic?

- Atopic dermatitis is associated with chronic systemic inflammation and increased cardiovascular risk.
- African American patients experience more severe disease and greater burden, yet their specific cardiovascular risk profile has not been well characterized.

## What does this study add?

- Identifies a distinct inflammatory cardiac profile in African American patients with atopic dermatitis, marked by elevated plasma biomarkers.
- Demonstrates higher prevalence of cardiovascular comorbidities, highlighting the importance of race-specific risk assessment.
